# Automatic migraine classification via feature selection committee and machine learning techniques over imaging and questionnaire data

**DOI:** 10.1186/s12911-017-0434-4

**Published:** 2017-04-13

**Authors:** Yolanda Garcia-Chimeno, Begonya Garcia-Zapirain, Marian Gomez-Beldarrain, Begonya Fernandez-Ruanova, Juan Carlos Garcia-Monco

**Affiliations:** 1 DeustoTech - Fundacion Deusto, Avda. Universidades, 24, Bilbao, 48007 Spain; 2 Facultad IngenieriaUniversidad de Deusto, Avda. Universidades, 24, Bilbao, 48007 Spain; 3 Service of Neurology Hospital de Galdakao-Usansolo, Barrio Labeaga, S/N, Galdakao, 48960 Spain; 4 Research and Innovation Department, Magnetic Resonance Imaging Unit, OSATEK, Alameda Urquijo, 36, Bilbao, 48011 Spain

**Keywords:** Feature selection, Classification, DTI, SVM, Boosting(adaboost), Naive bayes, Migraine, Committee

## Abstract

**Background:**

Feature selection methods are commonly used to identify subsets of relevant features to facilitate the construction of models for classification, yet little is known about how feature selection methods perform in diffusion tensor images (DTIs). In this study, feature selection and machine learning classification methods were tested for the purpose of automating diagnosis of migraines using both DTIs and questionnaire answers related to emotion and cognition – factors that influence of pain perceptions.

**Methods:**

We select 52 adult subjects for the study divided into three groups: control group (15), subjects with sporadic migraine (19) and subjects with chronic migraine and medication overuse (18). These subjects underwent magnetic resonance with diffusion tensor to see white matter pathway integrity of the regions of interest involved in pain and emotion. The tests also gather data about pathology. The DTI images and test results were then introduced into feature selection algorithms (Gradient Tree Boosting, L1-based, Random Forest and Univariate) to reduce features of the first dataset and classification algorithms (SVM (Support Vector Machine), Boosting (Adaboost) and Naive Bayes) to perform a classification of migraine group. Moreover we implement a committee method to improve the classification accuracy based on feature selection algorithms.

**Results:**

When classifying the migraine group, the greatest improvements in accuracy were made using the proposed committee-based feature selection method. Using this approach, the accuracy of classification into three types improved from 67 to 93% when using the Naive Bayes classifier, from 90 to 95% with the support vector machine classifier, 93 to 94% in boosting. The features that were determined to be most useful for classification included are related with the pain, analgesics and left uncinate brain (connected with the pain and emotions).

**Conclusions:**

The proposed feature selection committee method improved the performance of migraine diagnosis classifiers compared to individual feature selection methods, producing a robust system that achieved over 90% accuracy in all classifiers. The results suggest that the proposed methods can be used to support specialists in the classification of migraines in patients undergoing magnetic resonance imaging.

## Background

Migraine is a common and disabling type of headache with a worldwide one-year prevalence of 17% among women and 7% among men, and an incidence peak in early to mid-adolescence [1, 2].

Migraine attacks are characterised by recurrent episodes of severe headache with accompanying symptoms of autonomic nervous system dysfunction. While most patients have an episodic form of variable frequency, a subset develops chronic migraine (CM), a condition characterised by headaches on 15 or more days per month and present in approximately 2% of the general population [3]. For unclear reasons, the episodic form evolves into chronic migraine at a rate of 2.5% per year, particularly in patients with obesity, low-income, medication overuse and a high attack rate [4].

Feature selection forms part of data mining techniques, in which we calculated the scores for each feature and by selecting those that obtained the best scores in which the threshold or to adjust the decision functions to make the selection more restrictive, or to adjust it to the set of data that one wishes to subject to study [5]. Many methods exist [6–10]. We make this selection using a criterion function which is the separability between the classes, whereby we need classification in order to make the selection so that the decision function may reveal the critical features that make the subjects belong to one class or another.

There are different criteria used to make the feature selection to assess the degree of importance that one feature may have in terms of the set [11], such as according to the information [12, 13], classification error [14], consistency [8, 15], dependency [9] and distance between classes.

As has been stated, there is an internal classification for the feature selection method, although to obtain quantitative data to establish whether the selection made using the method is correct, we use an external classification with the following classifiers: SVM (Support Vector Machine) [16, 17], Boosting (Adaboost) [18] and Naive Bayes [19]. The first-mentioned classifier requires learning to select sub-sample to train the machine, setting aside the other samples for the classifier’s own validation. SVM (Support Vector Machine) enables to adapt the kernel, this being the one that makes the separation transfer the samples to the feature area [20]. Boosting is an algorithm that makes use of several single classifiers that might not individually be used, owing to their high level of inaccuracy. However, using them as a whole makes it possible to construct a far more accurate classifier [21]. The last-mentioned classifier, Naive Bayes, enables a probabilistic approach to find the most likely hypothesis given a set of samples. Explicit probabilities are also calculated for each hypothesis [22].

The features used in this study to select them and subsequent classification of subjects to obtain via different questionnaires, which focus on items related to migraine pathologies and magnetic resonance images with diffusion tensor.

We make an attempt in this study to analyse the critical features to differentiate between subjects with sporadic migraine, chronic migraine and medication overuse, and control groups – to make a classification that prevents the doctor’s work from making a per-patient diagnosis, as well as preventing incorrect diagnoses.

To undertake the classification, we obtain values taken from DTI (Diffusion Tensor Imaging) images in addition to selecting specific questionnaires about migraine [23]. These types of image enable white matter pathway integrity to examine in a non-invasive way. From these images we obtain the following measurements: fractional anisotropy (FA), to measure the completeness of the white matter; mean diffusivity (MD), to measure the mean molecular movement and/or square displacement of the general molecules, irrespective of the directionality of the tissue; radial diffusivity (RD), referring to the diffusivity direction perpendicular to the main diffusion axis; and axial diffusivity (AD), which refers to the diffusivity across the main axis, this being associated with the axon diameter [24, 25]. In this study the authors only use FA values from DTI images.

The main goal of this study is to automate the diagnosis of patients with migraine through the classification of features obtained from DTI images and psychological tests applied to patients. In this way, we can make a diagnosis about the person has migraine or hard headache, thus it helps to the doctors and supports their decisions when giving the prediction and their associated treatment. In addition to the classification itself, the feature selection aid to algorithms to decrease the number and specify the tests necessary to diagnose migraine and. In terms of DTI images, it can delimit the areas affected by this pathology. Regarding the tests used, specialized neuro-radiologists select and the choice of DTI images has been also decided by neuro-radiologists, because due to these images they observe the deterioration in the grey matter of the brain.

## Methods

### Participants

The sample used in this study comprises 52 subjects (15 control subjects, 19 patients with sporadic migraine and 18 with chronic migraine and medication overuse).

Table 1 shows the subjects used in the research, with the mean and typical deviation being shown for their age.

**Table 1:** Description of the sample

	N(%)	Chronic migraine	Episodic migraine	Controls	*p*-value
Total	52	18(34.6)	19(36.5)	15(28.9)	
Socio-demographic and clinical data					
Age	43.5(7.7)	43.8(7.1)	41.4(7.9)	45.7(6.8)	0.2
Sex (Female)	47(90.4)	14(77.8)	19(100)	14(93.3)	0.05
Cognitive reserve					
≤11(Low)	13(25)	9(50)	3(15.8)	1(6.7)	
(Low-Medium)	13(25)	4(22.2)	5(26.3)	4(26.7)	
16-18(Medium-High)	15(28.9)	2(11.1)	6(31.6)	7(46.7)	
>18(High)	11(21.2)	3(16.7)	5(26.3)	3(20)	

A psychologist conducted a depression test (Hamilton test) at the start of the study on patients with migraine [26], as well as a life quality test (SF-36) [27].

Once the selection of the patients and signed the informed consent form, the following tasks were then performed: firstly, a depression and anxiety test, followed by the MRI. They were given a migraine calendar on which to note down the migraine attacks they have from day-to-day, the measurement taken and the number of working days lost owing to migraine.

These would be assessed every 3 months by the neurology team to check whether the chronic migraine and drug abuse diminish with suitable treatment and/or whether patients with sporadic migraine end up resorting to medication overuse. They would be monitored for a year, after which time there would a last assessment by once again administering the depression and life quality tests.

All the information gathered would enable patients with migraine to classify according they have had a good prognosis (sporadic sufferers who continue to have this type of migraine or those who no longer resort to medication overuse) or a bad one (those who persistently resort to medication overuse or sporadic sufferers who no resort to medication overuse).

All the subjects with migraine included in the study attended quarterly consultations with their doctor so that a set of accumulated variables that described what had occurred over the 3 months. In addition to these variables, those extracted from the tests carried out are also added.

This study received approval from the relevant ethical committee.

### Questionnaires

Different questionnaires related to emotion and cognition were selected. These two factors influence the perception of pain, and so as well as seeing to what extent the plan is impacting on the everyday life of an individual (life quality and dependency tests), the aim is to also ascertain the extent of pain suffered by patients (number of days with pain, amount of painkillers, MIDAS) and their emotional state (depression and anxiety tests) and cognitive level (IQ).

#### Intelligence quotient (IQ)

IQ was estimated by the Word Accentuation Test, which requires the pronunciation of 30 low-frequency Spanish words whose accents have been removed [28].

#### General quality of life (SF-36)

The SF-36 is a structured, self-reported questionnaire that includes 36 items measuring health status across eight domains [27]. The scoring system generates subscale scores for physical functioning (PF), role limitations due to physical problems (RP), bodily pain (BP), general health perceptions (GH), vitality (VT), social functioning (SF), role limitations due to emotional problems (RE) and mental health (MH). Two summary scores derive from the SF-36: the physical component (PF, RP, BP and GH) and the mental component (VT, SF, RE and MH).

#### Specific migraine quality of life questionnaire (MSQoL)

The MSQoL is a self-administered questionnaire that consists of 20 items, each of which is rated using a response scale with four categories (1 quite much to 4 not at all) grouped into three dimensions: avoidance (10 items), social relationships (six items) and feelings (four items). The scores were determined by adding the items for each domain. An overall score was also determined by adding the 20 items [29].

#### MIDAS

This questionnaire assesses headache-related disability [30]. Migraine patients answer five questions about the frequency (days) and duration of their headaches in the last three months, as well as how often these headaches limited their ability to participate in activities at work, at school, or at home.

#### Beck depression (BDI) and anxiety inventories (BAI)

We employed the Beck Depression (BDI) and Anxiety Inventories (BAI) [31]. These questionnaires consist of 21 self-administered items about how the patient has been feeling in the last week. Each question has a set of at least four possible choices ranging in intensity.

#### Medication-dependence questionnaire in headache (MDQ-H)

Medication dependence was assessed by the medication-dependence questionnaire in headache patients (MDQ-H) [32]. MDQ-H consists of a 21-item, self-administered questionnaire inquiring about the number of units of treatment taken per week, the number of days of headache, and emotional distress related to the lack of medication, among others. For each item, subjects are asked to describe their medication consumption according to a seven-point Likert scale (1: never or not at all; 3: sometimes or a little; 5: often or quite a lot; and 7: always or completely). Total score was obtained by adding the scores for all the items. A high score means an important disturbance in the way the patient uses his or her medication.

All the questionnaires used in this study were validated in Spanish except for the MDQ-H (validated in French and English).

### Feature selection

#### Gradient tree boosting

Gradient Tree Boosting or Gradient Boosted Regression Trees (GBRT) refers to an automatic learning technique used in regression and classification models [33]. This algorithm forms a set of decision tree prediction models, whereby we construc the model in stages. This enables to optimize the arbitrary differentiable loss function.

Once the algorithms complete the classification, the *fit* method is then applied to adjust the model to the classification, followed by the transform method, which enables to confine the entry reduce feature set to the most important ones.

Thus, we obtain the reduced dataset when applying these methods, which will insert in the SVM classifier.

#### L1-based feature selection

Feature selection using the penalty L1 penalty is efficient when applied to spurious features mixed with relevant features [34].

The L1 penalty efficiency has proved when selecting features of scattered models, which makes it robust regarding features that have a major noise load. Therefore, the L1 method provides a range of solutions by inherently selecting features.

On this occasion, classification takes the form of the linear Support Vector Classification, which is a more flexible type of classification when selecting penalties and loss functions, as well as better scaling a greater number of existing samples. Moreover, as the number of samples is greater than the number of features, this selects the algorithm in order to resolve the problem with optimisation.

The algorithm apply *fit transform* method to this classification, which first adjusts the classification model and then reduces the entry reduce feature set according to the most important ones in terms of this classification.

One it obtain the reduce feature set, classification is then made using the SVM machine learning algorithm.

#### Random forest

In the case of Random Forest methods, each tree in the set – both in terms of classification and regression – is constructed from an extracted sample, i.e. a first sample from the training set [35]. When it divides a node while constructing the tree, the division selected is not the best one from among all the features. In contrast, the division obtained is nonetheless the best from among a random subset of said features.

Random forest is an estimator adjusted to a number of tree classifiers in several data set sub-samples by using the mean to improve accuracy of the prediction and control of any “over-fitting”. The size of the sub-sample is always the same as the original size of the entry initial full dataset.

When applying this method, the classification is first carried out and then it apply the *fit* and *transform* methods to the resulting model, by means of which Random Forest adjust the model and obtain the dataset solely with the most important features.

#### Univariate features selection

Within the univariate type of feature selection exist different methods, such as: “best K”, in which it disregard all features except the best “k” ones (the user decide this value), “percentile”, in which disregard all except a specific percentage of features issued by the user; using statistical tests such as the “false positive rate”, “false discovery rate” and “family wise error”; lastly, there is the method that enables you to fully administer the univariate method by selecting its own configuration, whereby the algorithm can select the best strategy with a range of hyper parameters [36].

The selection of “best K” method for this case study from among the univariate feature selections available, whereby the user may select the exact number of features with which they wish to subsequently carry out classification by disregarding the others that form part of the initial set of data.

Once it carries out the procedure, then applies *fit transform* method, which enables to adjust the model and the initial full dataset converted with all the features into a new set of data comprising only the most important ones.

### Classification

To check whether the feature selection has been effective, a classification carries out with the new set of data obtained via each feature selection method.

Taking into account that in this study, the authors proceed to classify 3 groups: control group, sporadic migraine and chronic migraine. Therefore, we use multi-class classifiers instead of binary classifiers.

#### SVM

The supervised classifier Support Vector Machine (SVM) is effective in higher-dimensional spaces and in cases where the number of dimensions is greater than the number of samples [37]. Regarding its decision function, it uses a subset of training points, meaning the classifier’s memory is efficient. SVM is also versatile, containing the option of selecting different kernel functions for the decision function. There are already a number of common specified kernels, although it is possible to personalise the kernel.

Moreover, we use cross-validation methods to ensure independence of the division between training and validation data, specifically the *Stratified K fold* method, which divides the samples into stratified folds, two training and validation groups according to the total number of elements. Each set group contains approximately the same percentage of samples of each target class as the complete set. The “folds” value is 8, i.e. the iterations made in the course of validation. Therefore, it produces a classification for each iteration and the final accuracy is the mean of these iterations.

This classifier allows multi-class classification according to a one-vs-one scheme.

#### Boosting (Adaboost)

This algorithm proposes an iterative training a series of base classifiers, whereby each new classifier may pay closer attention to any data that has been erroneously classified by previous classifiers in such a way to obtain a general classifier with high performance features when combine the aforementioned classifiers. For this, it creates series of iterations in which the classifier assigns a train output weight by adding it to the set to ensure to obtain the system’s global output via a weighted linear combination of all the base classifiers.

Therefore, it combines the predictions made by each base classifier via a weighted majority vote, thus generating the final prediction. It applies weights for each iteration to all the data and to the training samples. In the first iteration, all the weights applied are equal, whereby supplied the classifier with the original data. For each successive iteration, it modifies the sample weights individually and the algorithm is again applied to the “re-weighted” data. At a specific point in the classification, it applies greater weight to the training examples that have been erroneously predicted, where will assign the correctly predicted samples [38]. As the iterations advance, the samples that are difficult to predict receive increasing influence, enabling the algorithm to in turn be increasingly adjusted to the samples and thus obtaining the optimum classification.

In this classifier, it uses a decision tree inside the Boosting classifier parameters to improve prediction accuracy on a multi-class classification, instead the binary classification.

#### Naive bayes

Bayesian learning algorithms are able to calculate explicit probabilities for each hypothesis based on Bayes’ theorem, as well as being comparable to neural network methods and decision trees [39]. This algorithm enables a probabilistic approach to the inference based on the assumption that any unknown ones of interest remain probabilistic distributions. Therefore, it can achieve an optimum solution via such forms of distribution and the data observed, enabling the possibility of a hypothesis occurring to be quantitatively weighted.

This classifier has various features, among which each training sample affects the hypothesis probability, making the classifier more effective by being able to directly disregard any incompatible hypotheses. It associates a confidence percentage with the predictions by combining these predictions based on their confidence score. Moreover, it classifies every new instance as a prediction function of multiple hypotheses that are weighted by their probabilities.

Certain knowledge may also be included beforehand, i.e. even though not included at the start, the probability of each hypothesis and probability distribution of the samples must be estimated, for which purpose it takes the relevant information from the training phase. Naive Bayes can be applied in cases where there are a large number of medium-sized or large training sets, and where the features that describe the samples are independent of each other – as well as being commonly and successfully applied in diagnoses.

Specifically, this classifier implements the Gaussian Naive Bayes for classification that allows the multi-class classification to comply with the 3 groups in this study.

### Integration

**Fig. 1:**
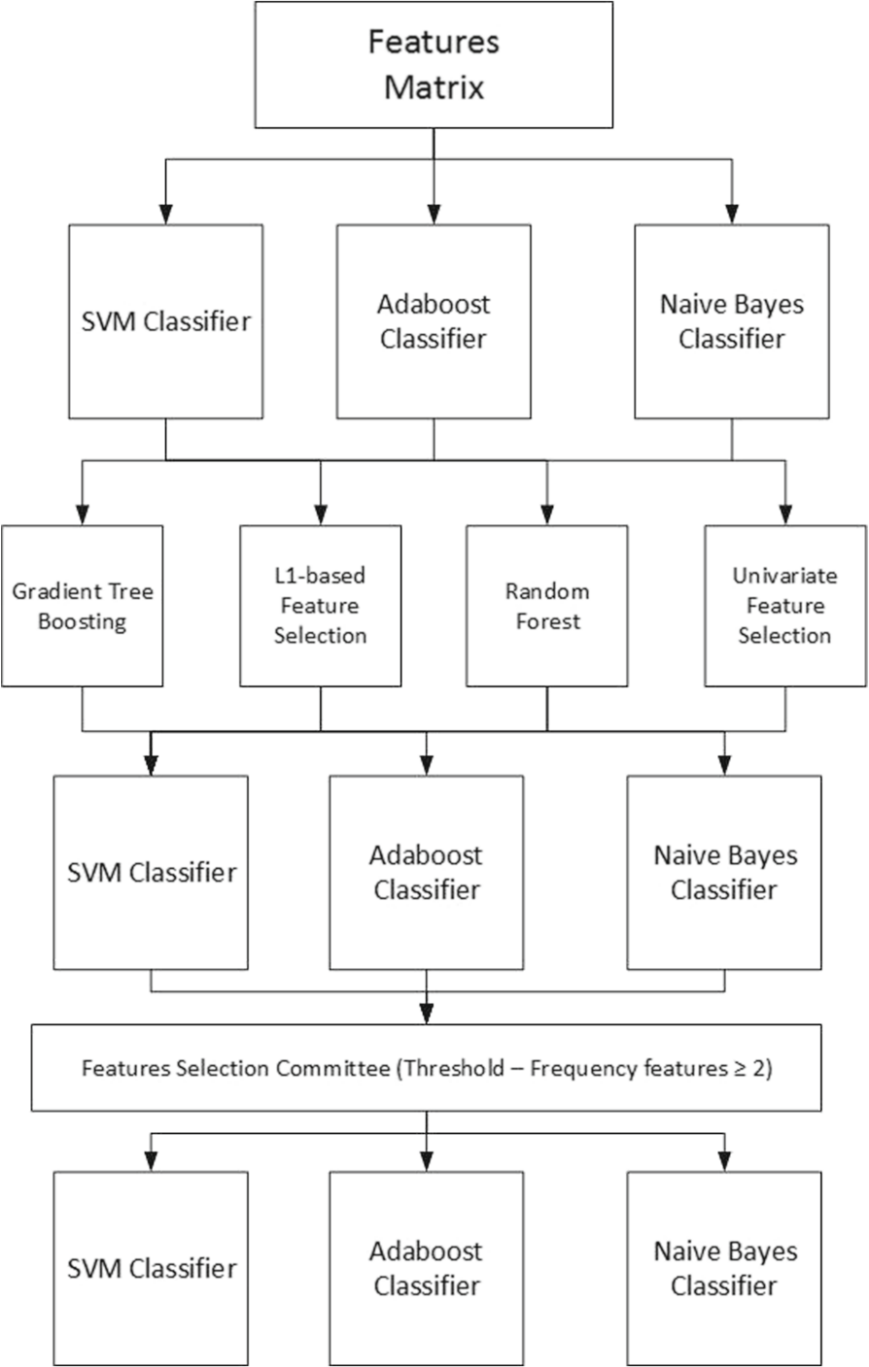
Block diagram showing the feature selection process and classification. Each of the selection methods is applied to the initial full dataset with all features, classifying them so as to quantify whether any improvement exists. The feature selection method committee is then applied and the resulting reduce set is once again classified

We perform some algorithms this study which integrates the selection of important features and classification. The process carried out for the study to ascertain what the most important features are with regard to differentiation of sporadic and chronic migraine pathologies and medication overuse, and their subsequent classification (Fig. 1), involves firstly introducing the initial full dataset with the samples and all the features associated with the different feature selection methods. The dataset resulting from each method contains all the features selected, and will introduce into the three classifiers: SVM, Boosting and Naive Bayes. From this step we will obtain the accuracy associated with each feature selection method.

The next step is to bring together all the resulting dataset and obtain the repetition frequency for each feature in these dataset. When carrying out the study using 4 methods, we thought that those features should be selected which are at least repeated 2 or more times from the 4 existing methods, whereby the chosen threshold is 2. This new reduce dataset is then obtained with all the samples together with the features obtained using the feature selection method committee [40, 41]. Thus, not only does this respond to the decision of a single method, but it is also necessary for at least two of the feature method reductions to coincide in the most important features.

Lastly, the final step involves classification of this new reduced dataset, whereby due to the datum that provides the accuracy, we may observe whether these methods improve the efficiency of the classifiers by themselves and with the committee.

All experiments were performed using the Python Scikit-Learn 0.17 (stable) version.

## Results

Several phases will need to complete in order to ascertain whether the feature selection methods improve classification. Firstly, we classify the initial full dataset with all the features. Feature selection will then be carried out using each of the 4 methods, obtaining a new reduce dataset for each of them. These new dataset will be again introduced into the classifiers, producing new accuracy. We will already be able to observe in this step whether the methods improve or worsen efficiency by themselves.

We create a method committee with a threshold of 2, including only the features repeat 4 or more times in a new reduce set. Thus, it is possible to ensure that not only are the features selected by a single method, but that there are at least 2 selected by taking into account the opinion of the methods used. And lastly this final dataset is then classified, obtaining the accuracy.

There are 3 groups to classify that belong to subjects who suffer from sporadic and chronic migraine pathologies and medication overuse, and the control group. These will respond to specific psychological tests, thus obtaining items from each test that are subsequently used for classification purposes.

### Classification with initial dataset

We obtain 90% accuracy when introduce all the features referring to sporadic and chronic migraine pathologies and medical overuse. Moreover the precision achieve is 85%, recall 91% and F1score 87%.

We achieve 93% accuracy in the classification of the three groups in the case of the Boosting classifier. The precision is 100%, recall 89% and F1score 93%.

Lastly, we obtain 67% accuracy using the Naive Bayes classifier (Table 2). The precision is 66%, recall 76% and F1score 69%.

**Table 2:** Accuracy, precision, recall and F1score of the initial full set with all features using the SVM, Boosting (Adaboost) and Naive Bayes classifiers

	Accuracy	Precision	Recall	F1score
SVM	90%	85%	91%	87%
Boosting (Adaboost)	93%	100%	89%	93%
Naive Bayes	67%	66%	76%	69%

### Feature selection methods

We select the most important features applying the methods according to the features of each method, i.e. according to a decision, variance function or feature correlation.

Table 3 shows the number of features that each method considers the most important, as well as the percentage decrease. The least restrictive method proved is L1-based feature selection, in which 20 features were disregarded, while the most restrictive was gradient tree boosting, which disregarded 37 of the 41 features. The following section shows the classification accuracy with the most important features according to each method.

**Table 3:** Number of remaining features and percentage decrease according to the number of initial features, applying this using all the feature selection methods

	Number of features	Percentage decrease
Gradient Tree-Boosting	4	90%
L1-based	21	48%
Random Forest	12	70%
Univariate	10	75%

### Classification using feature selection methods

As observed, each method selected certain features as being the most important. Table 4 shows the accuracy with the SVM, Boosting and Naive Bayes classifiers.

**Table 4:** Accuracy of the resulting dataset after having applied of the feature selection methods using the SVM, Boosting (Adaboost) and Naive Bayes classifiers

	Accuracy	Precision	Recall	F1score
SVM				
Gradient Tree Boosting	98%	100%	94%	96%
L1-based	88%	86%	85%	84%
Random Forest	91%	91%	85%	87%
Univariate	78%	80%	78%	78%
Boosting (Adaboost)				
Gradient Tree Boosting	94%	100%	92%	9%
L1-based	91%	91%	91%	89%
Random Forest	95%	100%	94%	96%
Univariate	87%	87%	87%	85%
NaiveBayes				
Gradient Tree Boosting	98%	96%	100%	98%
L1-based	61%	60%	78%	67%
Random Forest	92%	86%	98%	91%
Univariate	60%	53%	87%	66%

We obtaine the worst classification using the univariate feature selection method and subsequent classification using Naive Bayes with a 60% accuracy (precision 53%, recall 87% and F1score 66%), while the best classification is the gradient tree boosting - again using the Naive Bayes classification and with 98% accuracy (precision 96%, recall 100% and F1score 98%).

Referring to Table 2, the *univariate feature selection* method worsened the classification percentage by 14% with all the features available. In contrast, the gradient tree boosting method improved the classification percentage by 8% with the features selected as being the most important.

### Classification using the feature selection method committee

Lastly, we perform a method committee obtaining the most important features from each feature selection method, i.e. the features most frequently found in the resulting dataset will be the ones selected to carry out a new classification. The committee is formed not only to ascertain the opinion of a particular method but also because other opinions exist, with the one that is most repeated winning.

To this end, a threshold value needs to state with which to ascertain the frequency beyond to select the features. In this case and bearing in mind there are 4 methods, the value will be at least 2 repeated features. This means that 2 or more will be the frequency required to select the most important features from those obtained using the methods.

In Table 5 can be seen the number of features that meet the threshold requirement, whereby those features will be the ones used for the new classification. These features refer to the amount of medication taken, scores on depression scales, number of years that the subject has suffered from the pathology, with subjects’ physical functions and mental health, and anxiety scales.

**Table 5:** Features selected from the chosen threshold applied to the resulting features of each feature selection method

	Frequency (All = 4)
Total Pain days	4
Total Analgesics	4
Score MSQoL	3
Left Uncinate	3
Left Cingulate Gyrus	2
Score MDQ-H	2
Total Pain Month 1	2

Classification with this new set of features was again carried out using SVM, obtaining 87% accuracy, 93% precision, 92% recall and 92% F1score (Table 6).

**Table 6:** Accuracy, precision, recall and F1score with the dataset resulting from the feature selection method committee using the SVM, Boosting (Adaboost) and Naive Bayes classifiers

	Accuracy (Threshold ≥ 2)	Precision	Recall	F1score
SVM	95%	93%	92%	92%
Boosting (Adaboost)	94%	96%	89%	92%
Naive Bayes	93%	90%	98%	92%

Lastly, by comparing the improvement in classification between this latest value and those obtained previously from the initial classification and that carried out once we applied the feature methods, as we can see in Table 6 that this classification using the selection method committee improves on the accuracy of what had previously been classified. The percentage improvement of the L1-based feature selection method is 61%, this being considered a significant improvement of the accuracy. However, we see from classification of the dataset obtained using this method, it did not obtain a high accuracy and the classification could even be considered random (54%).

Therefore, if we disregard this percentage improvement, we see that improvement over the initial full dataset with all the features is 12%.

## Discussion

Features were selected in this study to carry out a better classification when differentiating between subject who suffer from sporadic and chronic migraine pathologies and medication overuse.

From an initial full set comprising all the subjects and all the features belonging both to psychological questionnaires, data regarding days with pain, amount of painkillers and values obtained from DTI images, we obtained 90% classification accuracy between the three groups subject to study in the case of the SVM classifier, 93% in the case of Boosting and 67% in the case of Naive Bayes. Therefore, the SVM classifier offers an efficient method for classifying samples, as it has solid foundations in terms of statistical learning, thus enabling to optimize the decision function in the training process [42].

As regards the SVM and Boosting classifiers, we obtaine an accuracy in classification of at least 90%. There is no study in which machine learning techniques have been applied to classify the migraine pathology, although they applied in other studies to other pathologies, e.g. in the study conducted by Dyrba, in which the used machine learning techniques – specifically the SVM classifier for classification of subjects via DTI images belonging to Alzheimer’s disease [43]. On this occasion, we obtained 80.3% accuracy. We achieved a better percentage in this particular article, as not only was classification made with the features obtained from DTI images, but features from specific questionnaires for migraine were also added. The SVM classifier was used in another study by Ingalhalikar, although on this occasion for classification of schizophrenia – also via DTI images [44]. The authors obtained a 90.62% accuracy following a feature selection process. From what can be observed obtaining features from DTI images is a good method for making the classification, facilitating the specialist’s work without the need to visually review images of all the subjects to determine whether they suffer from the pathology or are healthy. Any unnecessary costs are thus avoided.

Additionally and as in the study, feature selection implies an improvement in the accuracy of classifiers [44]. If the features that describe subjects are independent of each other in terms of the migraine pathology, then classification percentages are higher reducing the number of features. In the case of the SVM and Boosting classifiers, this increase is fairly insignificant, as in both cases the accuracy is already above 90%. Yet in the case of the Naive Bayes classifier, we can see a major improvement in the classification of the individuals subject to study.

Despite these good results, it should be noted that the study does not have a large number of samples in the full dataset, having fewer than 20 subjects per group. Therefore, the overfitting problem can be given by the low number of samples for the training, and the classification algorithm can be adjusted to really specific features of the training data. Therefore, in the future lines will include the fact of increasing the number of samples to avoid these problems and the main goal will bet the percentage of accuracy of the classifier.

The authors have used the same questionnaires and clinical assessments for machine learning to use before for establishing the clinical diagnosis. This fact could maybe introduce some circularity that the algorithms will certainly find these features to best differentiate the subject groups.

Due to the small number of participants, the testing was undertaken in the same dataset used for training and no holdout test set was used. For this reason, the performance measures may not generalise to unseen data.

Consequently, the conclusion can also be drawn that there are features which influence the classifiers, meaning that accuracy may be lower when introducing all the features into the classifier. We carried out a feature selection using a range of methods in order to conduct a robust study. As in the case of the studies [45, 46], several methods were chosen and a comparison made with dataset containing the original features, improving the classification in nearly all cases when applying such methods. When creating the feature selection committee and disregarding those features that did not meet the threshold, improving accuracy in the case of the three classifiers. Hence it can be shown that features existed throughout the dataset that either had no necessary importance for the purpose of making a proper classification or because they were deemed redundant. Although the accuracy obtained via the committee may not be the highest and were obtained specifically by a feature selection algorithm such as gradient tree boosting, the feature selection committee is deemed to achieve a more robust system as the best selection features from among four feature selection algorithms – not taking into account the opinion of just one of them.

The fact should also be taken into account that combining features deriving from DTI images and features obtained from specific questionnaires about this pathology provides a robust and reliable classification. In other words, the DTI images and questionnaires complement each other, the fact of having the two parts being deemed clinically vital to be able to establishing a result as to whether the subject suffers from migraine or otherwise. This occurs in the study carried out by Robinson [47], in which he makes use of magnetic resonance images and data from questionnaires to predict clinic aided tracheal intubation using multiple features.

## Conclusion

Distinguishing between patients with sporadic migraine, chronic migraine, and patients at risk of medication overuse is possible via feature selection techniques and machine learning. We obtained a classification with over 93% accuracy in the case of the three classifiers included in this article (SVM, Boosting and Naive Bayes). Moreover, the initial dataset containing 41 features (questionnaires and DTI images) was reduced when carrying out the feature selection to the 7 most important features with a combination of DTI images and questionnaires related to emotion and cognition. The classification ratio was improved by 28% in the case of the Naive Bayes classification due to this feature selection.

Thus, the method can classify patients due to a small set of features from specific questionnaires related to emotion and cognition, combined with features obtained from DTI images resulting from prior selection of the most important features.

This feature selection is effective when disregarding features that are deemed not relevant within the set, or those that are deemed redundant. In this way, the accuracy of the classifier improves when applying these types of method, obtaining a more robust and accurate system and preventing any confusion owing to features that are unimportant – achieving the best possible classification of samples with the best features.
